# Impact of Growing Up with a Chronically Ill Sibling on Well Siblings' Late Adolescent Functioning

**DOI:** 10.5402/2013/737356

**Published:** 2013-01-28

**Authors:** Sasha A. Fleary, Robert W. Heffer

**Affiliations:** Department of Psychology, Texas A&M University, MS 4235, College Station, TX 77843, USA

## Abstract

The purpose of this study was to explore the continuing impact of growing up with an ill sibling on well siblings' late adolescent functioning. Forty late adolescents (*M*_age_ = 18.78, SD = 0.83), who identified themselves as growing up with an ill sibling, completed a semistructured interview, demographic questionnaire, Personality Assessment Screener, and My Feelings and Concerns Sibling Questionnaire. Participants reported clinically significant problems on some PAS scales, and gender differences were found for acting out and alienation. Significant relationships were reported for communication and social withdrawal and alienation. Both positive and negative themes about the experience were elicited from the responses in the semistructured interview. This study provides evidence for some lingering negative effects of growing up with an ill sibling on well siblings' late adolescent functioning. Additionally, evidence for siblings' development of positive characteristics that may act as protective variables as they face the stressors of late adolescence was also highlighted.

## 1. Introduction

Adjustment and coping of children with pediatric illness has been extensively researched, with some emphasis placed on family adjustment and the reciprocal influence it has on ill children's adjustment and coping [1–3]. The majority of studies conducted on family adjustment have focused on the adjustment of parents [4–6]. Fewer studies have focused on the adjustment of siblings [7–10], and most of these studies are specific to siblings of children with pediatric cancer. 

Regardless of the severity of the pediatric chronic illness, siblings usually experience some emotional instability and disruption in their lives due to the diagnosis [11–14]. Breyer and colleagues [7] found that 59% of children showed new externalizing symptoms and 26% showed new internalizing symptoms following their siblings' cancer diagnosis. Siblings are also exposed to unique situations that other children are not privy to, which may have positive or negative impacts on well siblings' immediate and future well-being. Specifically, siblings may experience positive behavior changes including greater maturity, empathy, and compassion. This new-found empathy and compassion may often lead to a desire to help and take care of others [15]. Siblings' experiences with caring for and observing their ill siblings' course of illness may also give them an appreciation for life and understanding of its fragility that siblings in well families may not be able to comprehend [16, 17]. 

Researchers disagree on the pervasiveness of the negative impact of the diagnosis on well siblings' well-being. Specifically, Houtzager and colleagues [8] conducted a longitudinal study on siblings (7 to 19 years) of children diagnosed with cancer, following them up to 24 months after the cancer diagnosis, and found that siblings were most affected in the first few months following the diagnosis. Conversely, Alderfer et al. [18], in their study on adolescent siblings of childhood cancer survivors, found that siblings had elevated levels of posttraumatic stress when compared to nonaffected adolescents, suggesting that there may be some residual effects of the diagnosis on well siblings' functioning. 

To date, no research has explored the continuing impact of growing up with an ill sibling, on well sibling's late adolescent functioning. Individuals between the ages of 17 and 24 years are typically categorized as late adolescents/early adults [19]. These individuals face stressors and changes that could be challenging, including managing independence, leaving home for college and being separated from their immediate family, starting serious romantic relationships, and new responsibilities [19]. 

Siblings of children with chronic illnesses may have childhood experiences that could affect their adjustment to at least one of these stressors. Siblings may have been separated from their families for extended periods of time due to their ill siblings' hospitalizations [17]. They may also have had to exercise some independence due to parents having to spend extended periods of time caring for their ill sibling or them taking on a caretaker role for younger well siblings at an early age [15]. One can argue, therefore, that siblings of chronically ill children may have a unique *advantage* as they transition from adolescence to late adolescence, because of their childhood experiences.

Researchers have measured young adulthood adjustment of children with chronic illnesses and have found that these children tended to grow up to be more resilient adults, than those with a typical childhood experience, although others have found that some young adults display posttraumatic stress symptoms [20–22]. The extent to which well siblings' functioning mirror these findings are yet unknown.

## 2. Present Study

 Sibling relationships are believed to be one of the most powerful, extensive relationships in one's lifetime and are distinguished by its love, jealousy, companionship, compassion, and rivalry [23, 24]. This relationship is greatly affected when one sibling becomes seriously ill. For example, well children report feeling less close to their ill sibling [7]. Siblings of ill children have also reported high levels of anxiety and isolation [11, 13], envy, and contradictory feelings of guilt and resentment [12]. Some studies also found that well siblings have more difficulty adjusting and adapting to the illness than their ill siblings [14]. The purpose of this study was to explore the continuing impact of growing up with an ill sibling on well siblings' late adolescent functioning. By exploring well siblings' psychological functioning in relation to community patterns and how they perceive their experience as influencing their lives currently, the researchers hope to provide a clearer picture on well siblings' late adolescent functioning. Additionally, by retrospectively exploring well siblings' feelings and concerns about their ill siblings' diagnosis, the researchers aim to provide insight on how these variables impact well siblings' present functioning. 

## 3. Method

### 3.1. Participants

Participants (*n* = 40) were randomly selected from enrollees in a university psychology subject pool who identified themselves as growing up with an ill sibling during prescreening. They were between the ages of 18 and 21 years (*M*_age_ = 18.78, SD = 0.83), and most were freshmen (67.5%) at the university. Participants were predominantly Caucasian (75%) and females (60%). The majority of participants reported living in nuclear (77.5%) and step-parent (12.5%) families at the time of their siblings' diagnosis. Place in the family in relation to ill sibling was evenly distributed with 50% reporting being older and 50% reporting being younger than their ill sibling. All fathers of participants worked full time, while they were growing up, while 52.5% of mothers worked full time. The majority of mothers (87.5%) and fathers (82.5%) of participants had at least some college education. Participants varied in the diagnosis of their ill siblings.

### 3.2. Measures

#### 3.2.1. Demographics

Participants completed a demographic questionnaire that elicited information about themselves and their families. Specifically, questions were asked about family composition, sibling's diagnosis, caretaker arrangements, and parent education and work status.

#### 3.2.2. Psychological Functioning

The Personality Assessment Screener [25] (PAS) was used to assess for behavioral and emotional problems of potential clinical significance. The PAS is a 22-item measure that assesses a broad range of clinical problems and is comprised of the items proven to be maximally sensitive to clinical problems on the Personality Assessment Inventory [26] (PAI). The PAS yields a total score and 10 domain scores including negative affect, acting out, health problems, psychotic features, social withdrawal, hostile control, suicidal thinking, alienation, alcohol problems, and anger control. The PAS uses probability (*P*) scores, and the scores are interpreted as the probability that a person would obtain a problematic protocol if given the PAI. Therefore, any score above 50 *P* indicates that the person may be experiencing some clinical difficulty. 

#### 3.2.3. Sibling Perceptions

The “My Feelings and Concerns Sibling Questionnaire” [27] (MFCSQ) was adapted for this study. This 27-item questionnaire was originally designed to assess perception and adaptation of well siblings to pediatric cancer of a sibling and included four factors. The first factor is interpersonal (Cronbach *α* = 0.86), including nine items assessing siblings' perceptions of themselves in the experience. The second factor is intrapersonal (Cronbach *α* = 0.80), including seven items about how well siblings felt about their siblings' diagnosis. Factor 3 is communication (Cronbach *α* = 0.67), including four items that examined sibling's perception of their ability to communicate with parents and adults about the diagnosis. The last factor is fear of disease (Cronbach *α* = 0.65), consisting of three items that assessed siblings' fears and worries. Because the measure was designed for use specifically with school-aged siblings of children with cancer, the wording (replace cancer with illness) and instructions (think back to your childhood) were modified. Participants responded to all the statements on a 5-point Likert scale ranging from 1 = never to 5 = always. Higher scores were indicative of more negative perceptions.

#### 3.2.4. Semistructured Interview

Participants also participated in semistructured interviews. Questions were designed to elicit well siblings' perceptions of retrospective and current adaptation and coping with having an ill sibling. Responses to four of the questions were explored to meet the aims of this paper: “Sometimes good things come out of bad experiences. What would you say was good that came out of your sibling's illness?” “Looking back, what would you want to happen differently about the experience?” “What were your biggest fears then?” “How has the disease affected your life?”, and “How about now, do you have any fears or worries that stem from your sibling's illness?” Questions were developed based on information gathered from a review of the child literature on sibling coping and adaptation. 

### 3.3. Procedure

This study was approved by the Institutional Review Board at the author's university. Informed consent was obtained from all participants and all participants agreed to have their interviews audio-recorded. Participants were asked to complete the demographic questionnaire, MFCSQ, and PAS prior to the interview. Participants engaged in a 15-minute semistructured interview. The interviewer scanned the suicide items on the PAS prior to participants leaving, and a suicide risk assessment was conducted on those endorsing at least Slightly True on any of the items. The interviewer conducted a suicide risk assessment on a total of six participants. Participants were given credit for participation at the end of the interview and a debriefing form that included phone numbers through which they could seek psychological services if desired.

### 3.4. Statistical Analyses

 Specific analyses were conducted using SPSS PC 16.0 [28]. Descriptive statistics were initially calculated. Confirmatory factor analyses (CFA) were performed to determine how well the items on the MCFSQ yielded the same reliability coefficients reported by Carpenter and Sahler [27]. To explore well siblings' psychological functioning in relation to community norms, the PAS was hand-scored, and the scores were converted to *P* scores. These scores were compared to standardized community norms provided by the test author. Independent sample *t *tests were conducted to explore gender differences in PAS scores. To examine the relationship between well siblings' feelings and concerns about their siblings' diagnosis during childhood and their current functioning, Pearson correlations were computed between the PAS domain and total scores and the MCFSQ subscale scores. Thematic analyses were used to elicit themes about how siblings perceived growing up with an ill sibling as influencing their lives, both in the past and currently. 

## 4. Results

Descriptive statistics for the PAS are presented in Table 1. Three of the four factors on the MCFSQ produced similar reliability coefficients reported by Carpenter and Sahler [27] in their study on siblings of children with cancer (Interpersonal *α* = 0.89, Intrapersonal *α* = 0.75, and Communication *α* = 0.73). The Fear of Disease subscale was excluded from the study because it produced a very low reliability coefficient (*α* = 0.15). The low reliability of Fear of Disease may be attributed to this study's focus on a variety of chronic and terminal illnesses rather than solely cancer. 

Regarding well siblings' psychological functioning, well siblings displayed psychological problems in the range described in the PAS interpretive manual as being somewhat problematic but not necessarily of clinical significance for some domains (>60% of siblings had *P* scores in normal or mild range, see Table 1). For acting out, psychotic features, social withdrawal, and to a lesser extent hostile control, the distribution of *P* scores suggests that well siblings may be potentially experiencing clinically significant problems in these domains. The average total score, however, suggests that siblings are similar to well-adjusted community adults in their psychological functioning. When the scores were explored within the sibling sample, significant gender differences were found for acting out and alienation. Male siblings were significantly more likely to report behavioral problems associated with impulsivity, sensation seeking, drug use, or a combination compared to female siblings. Female siblings were significantly more likely to report failures of supportive relationships and a distrust or disinterest in such relationships. 

Pearson correlations revealed a positive relationship between well siblings' retrospective reporting of poor communication with parents during childhood and social withdrawal and alienation. Given the exploratory nature of this study, we noted that the relationship between alcohol problem and communication also approached significance. The PAS total score was positively related to retrospective reporting of interpersonal problems, that is, siblings' perceptions of themselves in the experience. 

Thematic analyses of siblings' responses to interview questions are presented in Tables 2, 3, and 4. Siblings identified greater awareness of illnesses (40%) and family bonding and support (100%) as good things that came from growing up with a sibling with a chronic illness. Well siblings identified their ill siblings' disease status, future, and their ability to help their siblings in the event of a crisis as fears they experienced while growing up. They also noted regretting not being more sensitive, jealous, and not spending enough time with family. The majority of siblings (62.5%) reported that the experience continues to affect their lives positively with cautiousness about own health, maturity, and appreciation of life being the most common positive themes. Approximately 17% of siblings noted that the experience continues to affect their lives negatively because they worry about the future and feel responsible for their siblings. Twenty percent of siblings reported that growing up with an ill sibling had no effect on their lives. When asked if they had any fears or worries now that stem from their siblings' illnesses, the majority of well siblings (60%) reported that they had no fear or worries while others reported fears about ill-siblings' health, siblings' death, disease worry, and own future.

## 5. Discussion

Results on well siblings' current psychological well-being were mixed and were somewhat contradictory. Examination of the mean scores in most domains suggested the possibility of siblings experiencing some problems, which may be expected given their developmental phase and the current stressors they may be experiencing in their lives such as adjusting to being freshmen in college. Noteworthy is that all of the mean scores with the exception of anger problem are in the lower part of the range suggested by the interpretive manual as being potentially problematic. Given the research on the psychological functioning of well siblings during childhood [7, 29], one would have expected these siblings to have much higher scores on the PAS especially in the negative affect domain, which is a measure of personal distress, and experiences of apprehension and unhappiness.

The results suggested that siblings may potentially experience clinically significant problems in acting out, which is indicative of behavioral problems. A significant gender difference emerged on this domain, with males having significantly higher scores than females. We speculate that these findings are due to males' tendency to act more impulsively and late adolescents' tendency to engage in more sensation-seeking behaviors and drug use on college campuses [30]. Also noteworthy is the gender difference for alienation. Females were more likely to report failure of supportive relationships and distrust in relationships. The gender differences in acting out and alienation may be reflective of the different coping styles of males (externalizing) and females (internalizing) in general [31, 32].

Social withdrawal on the PAS is characterized by social detachment and discomfort in close relationships, while hostile control is defined as an interpersonal style where there is a need for control and inflated self-image. The elevations on these two scales may be directly related to well-siblings' childhood experiences. Well siblings may have a history of being separated from their families for extended periods of time, and this may have contributed to the development of an avoidant attachment style. Being forced to understand the fragility of life at an early age [15] may also be responsible for them choosing to detach themselves from others. Regarding hostile control, researchers have found that well siblings learn responsibility from an early age, and in some instances they may have to adopt the caretaker role to their other siblings as well as their parents [17]. We hypothesize this early role reversal may potentially lead to a maladaptive manifestation of the behavior in late adolescence. Additionally, competition for parents' attention and affection during childhood may lead to siblings behaving in a manner to ensure they were attended to, and this too may be maladaptive in late adolescence.

Exploration of the relationship between retrospective accounts of feelings after the ill sibling was diagnosed and current psychological functioning resulted in some interesting findings with regard to communication. Both communication and social withdrawal and communication and alienation were positively related. Siblings who perceived their abilities to communicate with parents and adults about the diagnosis during childhood as poor were more likely to report social detachment and discomfort in social relationships and to report failure, distrust, or disinterest in supportive relationships. These findings can be interpreted in several ways. One explanation might be that siblings' may be inclined toward withdrawal in their communication style and interaction with others due to their temperament. Another potential explanation is that siblings may have learned to socially detach in response to not being able to communicate with parents and adults after the traumatic, life changing experience. A third explanation might be that parents may not have been forthright in their explanations about ill-siblings' diagnosis when talking to well siblings, and as a result well siblings learn not to trust others.

 A negative relationship was found between communication and alcohol problems. Siblings who perceived their abilities to communicate with parents and adults about the diagnosis as poor were somewhat less likely to report negative consequences related to alcohol use and abuse. One possible explanation for this might be that well siblings' tendency toward social withdrawal due to communication problems in childhood may mean that they do not engage in social activities that create opportunities for social drinking as frequently as others in their age. 

 A positive relationship was found between the interpersonal subscale and the PAS Total score. Siblings who had a more negative perception of themselves during the experience were more likely to have emotional and behavioral problems of clinical significance. This is consistent with the childhood adjustment literature; such that the more problematic and negative the experience was for the individual, the more the individual was at risk for emotional and behavioral problems [33–35]. Additionally, specific items on the interpersonal subscale assess parents' relationship with the well sibling and parents' and other adults' preoccupations with the ill sibling. High scores on this subscale, therefore, are indicative of parents' inability to meet the needs of the well sibling and failure to acknowledge their parental obligation to the well sibling despite having an ill sibling at home. These variables should be targets of intervention to reduce maladjustment and ineffective coping in well siblings.

The qualitative data gathered from siblings provided some insight on well siblings' adjustment in late adolescence. Specifically, the data suggest a high level of positive outcomes for siblings who grew up with an ill sibling. The development of characteristics such as appreciation of life, cautiousness about health, and maturity may act as protective variables for late adolescents in college who may be exposed to several opportunities for engaging in health risk behaviors. Empathy and compassion may also be helpful to well siblings as they engage in meaningful friendships and romantic relationships as well as when they transition to adulthood, in a number of important arenas.

Although participants reported negative experiences related to their siblings' illness, very few stated that they felt any lasting effect that continued on to college. Additionally, the majority of participants reported adapting to their family situation with no lasting hardship. This suggests that in spite of the potential for trauma from the experience, it may not necessarily result in posttraumatic stress. However, this is by no means uniform; adjustment may vary greatly between families, siblings, or even the same sibling, depending on life experiences at that particular circumstance. Participants who did report lasting effects were generally participants who lost a sibling to a terminal illness. 

Regret was the most coded negative response. While many participants stated that the change in their family dynamic was positive, others felt that the family became “more distant” or that the stress of medical visits and other illness-related appointments and events placed a large strain on the family system. This negative dynamic change was most evident when a sibling had a terminal illness.

## 6. Limitations and Future Directions

A limitation of this study is the use of a college sample. The participants in this study already proved that they were somewhat resilient based on their enrollment in college. It is possible that if a community sample was used, different results would be obtained and should be considered in future studies. The retrospective measurement of siblings' feelings and concerns regarding their siblings' diagnosis was another limitation of the study (i.e., retrospective responding compromises the accuracy of the responses). However, future studies are likely to use retrospective measurement due to the challenges of longitudinal research. In addition, the research questions of interest include how an older adolescent/young adult *looks back* reflectively on how earlier experiences have shaped current experiences and perceptions. A third limitation of the study was the use of the PAS as a measure of psychological functioning. The PAS is very brief, focuses more on psychopathology, and only gives the probability of a specific domain being problematic. A measure of both positive and negative psychological functionings may have been more suitable for this study. Future studies should measure positive and negative aspects of psychological functioning and also health-related quality of life. Future studies should also include a larger sample size with control group of late adolescent participants with which to compare standard measures of psychological functioning. 

## 7. Conclusions

This study provides qualitative evidence that several positive outcomes are related to having a sibling with a chronic illness, including greater health literacy awareness and higher levels of family cohesion. However, for a subset of participants (especially those whose sibling had a terminal illness), a number of negative outcomes emerged, including feelings of regret and fear and a breakdown in family communication and bonding. 

The present study also provides quantitative evidence for some lingering negative effects of growing up with an ill sibling on well siblings' late adolescent functioning during transition to adulthood. Specifically, well siblings' inability to communicate with parents and adults about their ill-siblings' diagnosis may have negative effects on their later social functioning, including their ability to be comfortable and trusting in social relationships. The results of the study also suggest that parents' ability to manage the needs of their well children is crucial to their children's resilience. Specifically, if parents focus their attention on the ill sibling at the expense of the well sibling, a higher probability exists that the well sibling would be susceptible to behavioral and emotional problems in late adolescence. Further research should explore how service providers may be able to increase protective factors and ameliorate risk factors, especially for families that may be at higher risk for negative adjustment outcomes.

## Figures and Tables

**Table 1:** Distribution of the PAS scores based on risk for problems and correlation with MFCSQ scores.

PAS Scales	PAS Score Classifications				Gender MD	Inter-personal *r*	Intra-personal *r*	Communication *r*
	Normal *N* (%)	Mild *N* (%)	Moderate *N* (%)	Marked *N* (%)				
Negative affect (*M* = 43.67, SD = 17.22)	24 (60)	0 (0)	12 (30)	4 (10)	1.43	0.15	0.13	0.12
Acting out (*M* = 51.74, SD = 20.15)	18 (45)	0 (0)	14 (35)	6 (15)	22.54***	0.15	0.10	−0.13
Health problems (*M* = 43.55, SD = 11.95)	21 (55.3)	10 (26.3)	6 (15.8)	1 (2.6)	−4.14	−0.06	0.12	0.27
Psychotic features (*M* = 52.16, SD = 16.78)	21 (53.8)	0 (0)	16 (41.1)	2 (5.1)	2.34	0.17	−0.10	−0.10
Social withdrawal (*M* = 65.09, SD = 19.74)	11 (29)	0 (0)	14 (36.8)	13 (34.2)	4.02	0.28^†^	0.20	0.37*
Hostile control (*M* = 51.25, SD = 5.57)	0 (0)	22 (59.5)	15 (40.5)	0 (0)	−1.17	0.01	−0.09	−0.27
Suicidal thinking (*M* = 45.13, SD = 16.22)	32 (82)	0 (0)	3 (7.7)	4 (10.3)	−2.02	−0.01	0.11	0.05
Alienation (*M* = 42.23, SD = 11.87)	19 (48.7)	14 (35.9)	6 (15.4)	0 (0)	−8.76**	0.20	0.02	0.32*
Alcohol problem (*M* = 43.01, SD = 10.88)	8 (20.4)	26 (66.7)	4 (10.3)	1 (2.6)	4.06	0.02	0.08	−0.30^†^
Anger control (*M* = 49.52, SD = 13.56)	12 (31.6)	17 (44.7)	8 (21.1)	1 (2.6)	4.57	−0.02	0.20	−0.10
Total score (*M* = 37.74, SD = 27.11)	12 (37.5)	9 (28.1)	9 (28.1)	2 (6.3)	6.56	0.43**	0.27	0.15

Note. MD: mean difference; PAS: Personality Assessment Screener.

^†^ *P* < 0.10, *P < 0.05, **P < 0.01, ***P ≤ 0.001.

**Table 2:** Siblings' responses to the question “How has the disease affected your life?”.

Theme	*N*	Quotation
Positive^a^		

Awareness	16	
Emotional support for others		“She had more strength to overcome different things. We'd just look at people and see if they had an illness, we would see past that and see the person.”
Health literacy/understanding		“I guess I'm more informed about what asthma is, and when other people have it I can sympathize with them. Not that I have it, but since I know what it is, I'm able to relate to people in that sense.”
Financial support/responsibility		“We donate to the leukemia fund now, so it's definitely raised my awareness of the disease.”
Sensitivity		“I'm more sensitive to it now, like when I hear “meningitis” it kind of snaps my neck and I'm like, whoa, what did you say? But then I have a testimony for it, in a small way.”
Family/bonding support	40	
Scheduling		“We had to have a pretty strict schedule, but it united us as a family, cause we'd work together to get the schedule done and help my mom and all that.”
Parental support		“My parents were really supportive, so that helped a lot.”
Respect for sibling		“This disease probably makes me appreciate my brother more; it's pretty amazing he's alive. We've gotten really close since I've left for school.”
Family teamwork		“It basically brought us closer together because we were constantly trying to take care of him, and not just one person could do all that. We all pitched in.”
Sensitivity		“I'd say we're a lot more receptive to grief. I'd say we're a lot more sensitive than other families are.”

Negative^b^		

Fear	17	
Heritability of illness		“Supposedly it's hereditary. But, like our kids wouldn't get it, but our grandkids might. So I guess that's a fear I have.”
Inability to aid Sibling		“My biggest fear was that they'd have an asthma attack and I wouldn't be able to do anything about it.”
Fear for sibling's future		“Just that he won't ever grow out if it. The doctors said he might, but then they said it's not like anything they've seen before, so that's kind of scary. That he could always be hurting. He doesn't know any difference.”

Regret^c^	27	

Time spent with parents/family		“I wish I had gotten to hang out with my parents more.”
Insensitivity to sibling		“I wish I wasn't so insensitive to her about it. That might offend her.”
Jealousy		“I sometimes got jealous of her, because she got more attention from our mom and dad.”
Change in family dynamic		“After she died, my dad has really changed, as far as showing any emotion. I think it hardened him as a person, and that's been the biggest change. Showing emotion is hard for him now.”

^a^“Sometimes good things come out of bad experiences. What would you say was good that came out of your sibling's illness?”

^b^“Looking back, what would you have want to happen differently about the experience?”

^c^“What were your biggest fears then?”.

**Table 3:** Siblings' responses to the question “How has the disease affected your life?”.

Theme	*N* (%)	Quotation
Positive	25 (62.5)	
Cautious about health	10 (25)	“It did make me like more health conscious”
Empathy/compassion	4 (10)	“…able to also relate with people that have siblings or who are struggling with illnesses … “Hey, I've been there and this is how, you know, I got through it, and you know there's a light at the end of the tunnel.”
Cautious about others health	2 (5)	“like-being-it made, it made me be more precautious…I am really like precautious of little kids…”
Accepting of others	2 (5)	“I'm more open to people… cause my sister would often tell me a lot of things…if she were to tell the people about it at school, they would like shy away from her…if people like tell me something about themselves, I'll just listen and accept them for the way they are.”
Aware of unpredictability of health	8 (20)	“…guess it just kinda also makes you realize that you know life can't be perfect and so that you know you can't expect everyone to have perfect health”
Maturity	9 (22.5)	“I think I am not as petty and I don't think of little things in a very big way and I don't overreact to situations the way a lot of teenage kids do”
Appreciate life	7 (17.5)	“it is made me really thankful for being healthy, like I think about that everyday”
Brought family closer	4 (10)	“I do wonder sometimes if our family would be as close as we are if it was not for…”
Knowledge	4 (10)	“… it is raised my awareness about the, about the disease…and um just kind of help others to be aware of it I guess too”
Reinforce religion	2 (5)	“guess the fact that uh, the doctors like gave him like a five percent chance and he pulled through…we are all like catholic so that's probably enforced that in my life”

Negative	7 (17.5)	
Overprotective parents	1 (2.5)	“My mom's a little over protective now”
Responsibility for sibling	3 (7.5)	“I think I probably a lot more protective over my little sister than I would have been”
Worry for the future	2 (5)	“It's made me think about having kids and how scary it is”
Paranoia about sickness	1 (2.5)	“…when I see them (others' children) running around and breathing a certain way, I'm like you should go check if they have asthma cause they probably do and one day you know, they will probably have an attack and you won't even know what to do and stuff.”
Indifferent	8 (20)*	
No effect	7 (17.5)	

*One person was indifferent because they were later diagnosed with the same disease.

**Table 4:** Siblings' responses to the question “How about now, do you have any fears or worries that stem from your sibling's illness?”.

Theme	*N* (%)	Quotation
No fears	24 (60)	
Sibling's health	8 (20)	“You know I probably worry for her more just knowing she only has one kidney and don't want to hurt that-don't want anything to happen to it”
Sibling's death	4 (10)	“Um, I'm afraid that he's gonna die, really a lot sooner than I think he should…and that's kinda hard, he's like one of my best friends”
Disease worry	4 (10)	“I guess I do kind of worry about like cancer, most people my age don't…but just in general I do”
Own future	5 (12.5)	“That maybe I'll have a child that will have problems, I think about that a lot”
